# *In vivo* magnetic resonance imaging of a mouse model of myelofibrosis

**DOI:** 10.1038/bcj.2016.97

**Published:** 2016-11-11

**Authors:** S Matsuura, S Patterson, H Lucero, O Leiva, A K Grant, V L M Herrera, K Ravid

**Affiliations:** 1 Department of Medicine and Whitaker Cardiovascular Institute, Boston University School of Medicine, Boston, MA, USA; 2 Department of Radiology, Beth Israel Deaconess Medical Center, and Harvard Medical School, Boston, MA, USA; 3 Department of Biochemistry, Boston University School of Medicine, Boston, MA, USA

Myelofibrosis is a slowly evolving pathology, characterized by increased myeloid cells and structural abnormality of the bone marrow matrix, which at end-stage manifests in excessive deposition of reticulin fibers and cross-linked collagen in the bone marrow, suppression of normal hematopoiesis and bone marrow failure.^1, 2^ Clinically, myelofibrosis occurs secondary to a variety of hematologic conditions,^1^ but is mostly associated with primary myelofibrosis (PMF).^3, 4^ Histological features of overt PMF include bone marrow with abundant reticulin/collagen fibers and later-stage osteosclerosis, although the pre-fibrotic stage mainly exhibits an abundance of megakaryocytes.

Although histopathology of bone marrow aspirates is the current standard for PMF diagnosis, magnetic resonance imaging (MRI) can provide non-invasive serial three-dimensional imaging of the bone marrow in contrast to restrictive clinical biopsy-dependent or murine end-stage conventional histology. Recognizing the potential of MRI to identify advanced myelofibrosis, a published study focused on T1-weighted MRI of bone marrow in overt-to-advanced PMF patients. Matching MRI images to biopsy–histologic studies has shown clinical feasibility of MRI, as a non-invasive method of determining extent of myelofibrosis combined with biopsy findings.^5^ Application of T1-weighted spin echo (T1-SE) and short T1-inversion recovery sequences showed a low-intensity signal (dark) in myelofibrotic marrow in femur and lumbar vertebrae that was distinguished from fat (light) on T1-SE.^5^ This confirmed a prior study detecting decreased T1-weighted MR-signal and increased T2-weighted signal in overt PMF, but not in essential thrombocythemia.^6^ These studies did not investigate pre-fibrotic early PMF.

Here we studied for the first time the potential of MRI to detect myelofibrosis in a mouse model of PMF, also asking whether this modality can capture early and late stages of the pathology. To this end, we performed fat-suppressed, 9.4-T T2-weighted rapid acquisition with refocused echoes (RARE) MRI of Gata-1^low^ knock-in mice (ΔneoΔHS mice originating from^7^ and available as mixed 129/C57BL/6 background^8^) at the pre-fibrotic stage<16-weeks old (wo), early myelofibrosis from 16–36 wo and overt myelofibrosis >36 wo (our observation, and as Varricchio *et al.*^9^). The general goal was to evaluate MRI as an experimental tool for assessing myelofibrosis in mice, with lessons learned on potential sources of the MRI signal in this pathology.

As shown in Figures 1a and d and Supplementary Figure S1, scant reticulin fibers are observed in 12-wo Gata-1^low^ mice, and none in wild type (WT) corresponding controls. Yet, T2-weighted MRI clearly distinguishes between 12-wo Gata-1^low^ and WT animals at pre-fibrotic stage, and 45-wo mice at myelofibrotic stage (Figure 1b). Bright T2-signals are detected in the femoral marrow of Gata-1^low^ mice that is largely absent in age-matched WT mice, except for the midline nutrient vessel (Figure 1b). The bright T2-MR signal was less homogeneous in 45-wo Gata-1^low^ femurs, compared with the 12-wo Gata-1^low^ femurs (Figure 1b), consistent with the appearance of osteosclerosis changes (bone yields a dark T2-MR signal) at 45 weeks (Figure 1c).

In another set of experiments, Gomori staining detected increasing reticulin fiber density in 20- to 40-wo Gata-1^low^ femurs, confirming myelofibrotic progression (Figure 1d). T2-weighted MRI also easily detected bright T2-signal in these Gata-1^low^ mouse femurs, compared with age-matched WT mice, which, again, only showed a filamentous structure along the longitudinal axis consistent with the nutrient vessel (Figure 1e). However, in both 40- and 45-wo Gata-1^low^ mice, the area occupied by the bright T2-MR signal was less homogeneous, compared with T2-MR signal at younger ages: 12-, 20- and 30-wo Gata-1^low^ mice, corresponding to osteosclerotic changes at 40 and 45 weeks (Figures 1c and f; see arrows). Development of osteosclerosis is consistent with late-stage PMF.^10^

Fat suppression in all T2-MRI eliminated fat as a confounder for increased (bright) T2-signal (Figures 1b and e). This was confirmed by comparing fat-suppressed images with non-fat-suppressed images, and by confirming the absence of a chemical-shift displacement artifact at low readout bandwidths^11^ (data not shown). In T2-weighted MR images, increased water/proton content, as in edema and increased cellularity, yield high (bright) MR-signal intensity. To elucidate what components of the Gata-1^low^ bone marrow might contribute to the bright T2-MR-signal, in addition to fiber deposition, we compared the cellularity and lineage profile of Gata-1^low^ versus WT femoral bone marrow. Bone marrow cellularity decreased with age in both WT and Gata-1^low^ mice, but age-matched comparisons show greater decrease in cellularity in Gata-1^low^ mice than in WT (Figure 2a). The reduced cellularity in Gata-1^low^ mice compared with age-matched controls is not consistent with the augmented T2-MR signals in Gata-1^low^ mice, thus suggesting another determining factor. However, lessened cellularity might underlie the difference in T2-MR signal intensity between 12-wo (brighter) and 45-wo (slightly dimmer) Gata-1^low^ T2-MRIs.

Lineage composition of bone marrow was unchanged with age in WT mice (Figure 2b). Interestingly, in pre-fibrotic young Gata-1^low^ animals, B lymphocytes (B220+) were increased by ~1.5-fold compared with WT animals, possibly indicating an underlying inflammatory process leading to edema. The percentage of these cells, however, decreased in old, fibrotic Gata-1^low^ mice compared with WT. Fluid collecting in the cavities (edema) could increase T2-MR signals. To probe this, mice were injected with Evans-blue dye to test for dye extravasation, as described Radu and Chernoff.^12^ Gata-1^low^ male mice (*n*=4) with advanced myelofibrosis (24-wo mice) exhibited a trend for increased dye extravasation compared with WT mice (*P*=0.45; data not shown).

Importantly, consistent with clinical early^13^ and overt^14^ PMF, megakaryocytes (CD41+ cells) were confirmed to be three- and sixfold increased in pre-fibrotic young, and advanced myelofibrotic old Gata-1^low^ animals, respectively, compared with age-matched WT mice. Of note, although megakaryocytes comprise <1% of bone marrow cells in number (Figure 2b), on histology (Figure 2c), the tissue area covered by recognizable, large megakaryocytes is typically 6–9% (Figure 2d). As a hallmark of both pre-fibrotic and myelofibrotic PMF, this suggests the intriguing hypothesis that megakaryocyte abundance could contribute to bright T2-MR signals within the bone marrow tissue.

Taken together, this is the first study evaluating *in vivo* T2-weighted MRI in a mouse model of myelofibrosis, with examination of potential sources of the MRI signal, and providing proof-of-concept that this non-invasive modality can detect pre-fibrotic stages in mice. It is intriguing to speculate that future pre-biopsy MRI of the human pathology might guide in some cases decisions on if and where to biopsy.

## Figures and Tables

**Figure 1:**
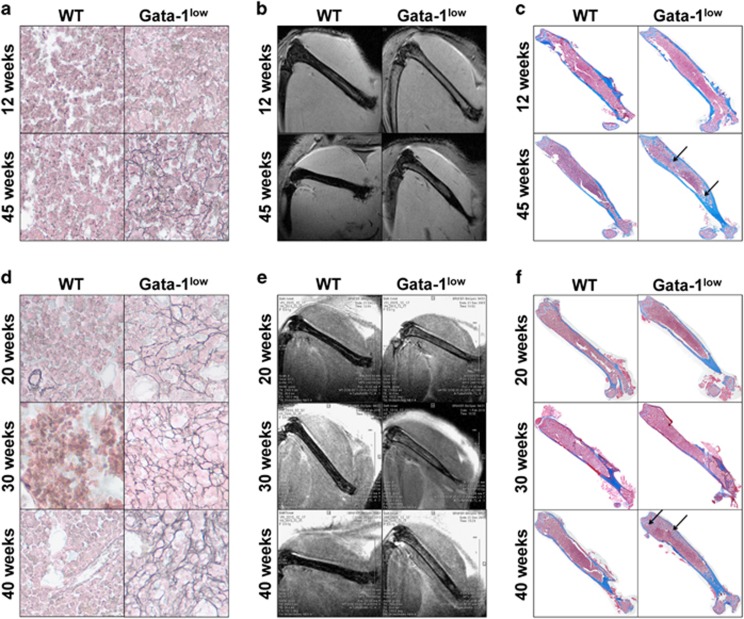
(**a**, **d**) Representative images of Gomori staining for reticulin fibers of femoral marrow of male wild-type (WT) and Gata-1^low^ mice at the indicated ages. Shown are images of areas representing the most reticulin fibers in each section (original magnification: × 400; additional representative images are in Supplementary Figure S1). (**b**, **e**) Representative images of *in vivo* 9.4-T T2-weighted MRI. Two-dimensional (2D) gradient-echo (TR=1000 ms, TE=2.3 ms) (**b**) and T2-weighted 2D fast spin-echo (TR=2500 ms, TE=30 ms) (**e**) of male WT and Gata-1^low^ mice at indicated ages; *n*=2−4 mice per age, including *ex vivo* MRI of femurs, which showed similar findings (not shown). (**c**, **f**) Representative composite images of Masson's trichrome-stained sections from femoral marrow corresponding to mice subjected to MRI in **b** and **e** (original magnification × 40). Blue staining denotes collagen; arrows indicate osteosclerotic areas.

**Figure 2:**
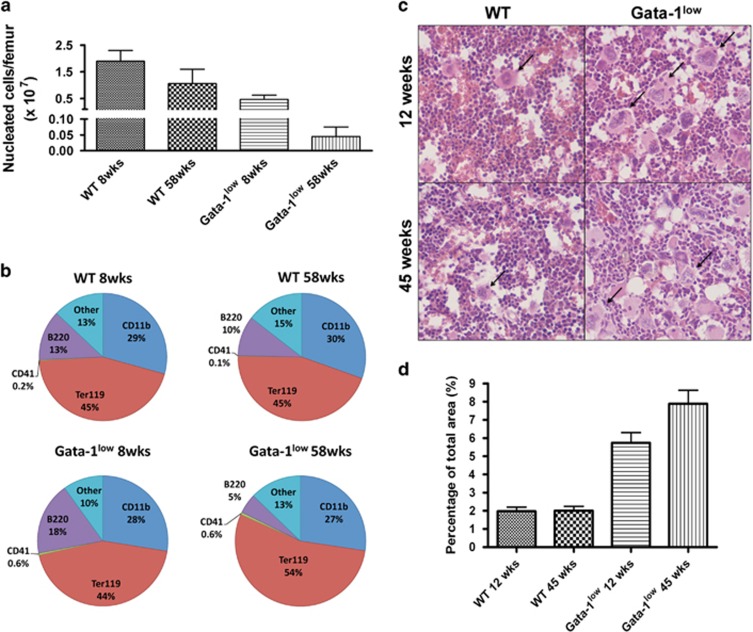
(**a**) Nucleated cell count per femur from 8- and 58-weeks old WT and Gata-1^low^ male mice, representing the extremes of the spectrum of myelofibrosis. Data shown are averages ±s.d. of three mice analyzed per group, except for the 58-week old Gata-1^low^ with two mice. Significant differences were observed between the young WT and young and old Gata-1^low^ mice (*P*<0.05). (**b**) Lineage composition of bone marrow of animals shown in **a**. Percentage of total bone marrow of B220+ B-lymphocytes, CD11b+ myeloid lineage cells, Ter119+ erythroid lineage cells and CD41+ megakaryocytes. ‘Others' denote percentage of cells negative for all markers tested. Numbers represent averages of the group. Statistically significant differences (*P*<0.01) were only seen in B220+ between young and old Gata-1^low^ mice. (**c**) Hematoxylin–eosin staining of bone marrow section from WT and Gata-1^low^ animals, at indicated ages. Shown are representative images of tissue sections derived from mice analyzed in **a**. Arrows point to typical megakaryocytes. (**d**) Measurement of area occupied by megakaryocytes in histological sections using Image J software analysis of samples as in **c** (original magnification × 40). Statistically significant differences (*P*<0.05) between WT and Gata-1^low^ mice were observed.
