# Supplementary material for: In vivo magnetic resonance imaging of a mouse model of myelofibrosis
Source: Blood Cancer J. 2016 Nov 11;6(11):e497–. doi: 10.1038/bcj.2016.97 (PMC5148061; doi:10.1038/bcj.2016.97)
Supplement: Supplemental Methods [file bcj201697x1.docx]

**Supplemental Information**

**Supplemental Methods**

Animals

Gata-1^low^ mice obtained as in[^1^](#_ENREF_1) were housed under the rules and guidelines of the IACUC (Institutional Animal Care and Use Committee) of the Boston University School of Medicine. All experiments and procedures involved in the manuscript were reviewed and approved by the IACUC (Institutional Animal Care and Use Committee) of the Boston University School of Medicine. C57BL6J animals were obtained from Jackson Laboratories (Bar Harbor, ME).

MRI

Imaging was performed using a horizontal-bore 9.4 Tesla scanner (Bruker Biospec 94/20, Billerica, MA).  Mice were anesthetized with isoflurane in oxygen and placed in the scanner with their femurs situated over a 20mm receive-only surface coil. Radiofrequency transmission was provided by a 84 mm quadrature volume coil. T2-weighted images were acquired using a RARE (rapid acquisition with refocused echoes) pulse sequence. Imaging parameters were TR = 2500 millisecond (msec), TE = 30 msec, RARE factor 8, 150µm in-plane resolution and 500µm slice thickness. To investigate T2* relaxation times, 2D multi-gradient echo images were acquired with TR=1000ms, TE ranging from 2.3 to 30ms, 30 degree tip angle, 150µm in-plane resolution and 500µm slice thickness.

Histology

Right femurs were collected and fixed in 4% paraformaldehyde, and sent to AML laboratories (Baltimore, MD) for decalcification, processing, embedding in paraffin, and sectioning in 3-5μm thick sections. For modified Gomori’s silver staining of reticulin, slides were heated to 60°C, deparaffinized and incubated in acidified 0.5% potassium permanganate (Scytek, West Logan, UT) for 5 minutes which produced a color change in the sections from clear to purple. The slides were washed with ddH_2_O and incubated in 5% oxalic acid (Sigma-Aldrich, St Louis, MO), until clear again. The slides were washed in distilled water and placed in ferric ammonium sulfate sensitizing solution (Scytek) for 10 minutes. The slides were washed in running tap water for 5 minutes and dipped in silver solution 14 times for 4 seconds per dip. The preparation of the silver solution involved making 20 mL of 10% silver nitrate solution (Scytek) in ddH_2_O in a 50 mL conical tube and adding ammonium hydroxide drop by drop until clear again followed by 20 mL of 3% sodium hydroxide. Ammonium hydroxide was added once again until a fine black precipitate remained in solution. The solution was poured into a 500 mL acid-washed beaker and made up to 200 mL with ddH2O.  The slides were dipped in three changes of ddH2O and reduced in 4% formaldehyde (catalog # BP531-25, Fisher) made in ddH2O until the tissue sections turned gray black. The slides were washed in ddH2O for 5 minutes and toned using 0.5% gold chloride (Scytek) for 1 minute, rinsed in distilled water, and fixed in 5% sodium thiosulfate solution (Scytek) for 1 minute followed by another wash in running tap water for 5 minutes. The slides were incubated in fast red stain (Scytek) for five minutes, dehydrated and cover slipped. Hematoxylin and eosin staining and Masson’s trichrome staining (Sigma Aldrich, St Louis, MO) were performed using routine staining methods available elsewhere. Composite images of Masson’s trichrome staining were generated using Photoshop (Adobe, San Jose, CA). Images were acquired using a Nikon bright field Eclipse 50i microscope and a Nikon Plan 4x and 40x objective (Nikon, Japan) equipped with a SPOT Insight2 camera and SPOT imaging software 5.0 (SPOT Imaging, Sterling Heights, MI).

Bone marrow nucleated cell count and flow cytometry

One femur per animal was used. Bone marrow was flushed in a buffer containing 0.5% BSA and 1 mM EDTA in phosphate buffered saline (PBS). An aliquot of cells were lysed in 17 mM Tris, 140 mM NH4Cl at pH 7.2 and used for nucleated bone marrow cell count by hemocytometer. The remaining cells were used for analysis of lineage markers. CD11b PE-Cy7 (M1/70, dilution 1:400), B220 APC (RA3-6B2, dilution 1:200) (eBioscience, San Diego, CA), Ter-119 PE (TER-119, dilution 1:200), CD41 FITC (MWReg30, dilution 1:100) (BD Biosciences, San Jose, CA) and zombie aqua (dilution 1:100) (Biolegend, San Diego, CA) were added to bone marrow cells and incubated for 15 minutes at room temperature and analyzed on a LSRII flow cytometer using FACS Diva software (BD Biosciences).

Evans blue extravasation assay

Animals were injected with a 200 μl solution of 0.5% sterile solution of Evans blue in saline. After 30 minutes femur bones were collected and fixed in 4% PFA. One femur was weighted, cut at the shaft and incubated for 48h in 500 μl of 4% PFA at 55°C. The PFA solution was transferred to a new tube and centrifuged to pellet any tissue debris present in the supernatant. The absorbance of the PFA solution at 610 nm was recorded, and the concentration of Evans blue in the solution was calculated based on a standard curve. The amount of Evans blue per femur was normalized to the weight of bone[^2^](#_ENREF_2).

1. Eliades A, Papadantonakis N, Bhupatiraju A, et al. Control of megakaryocyte expansion and bone marrow fibrosis by lysyl oxidase. *J Biol Chem*. 2011;286(31):27630-27638.

2. Radu M, Chernoff J. An in vivo assay to test blood vessel permeability. *J Vis Exp*. 2013(73):e50062.

**Supplemental Figures**

Figure S1: Additional representative images of Gomori’s silver staining of reticulin in bone marrow sections from Gata-1^low^ animals at the indicated ages.
